# Clinico-Dermoscopic Findings of Jacquet’s Erosive Dermatitis in Adults: A Report of Two Cases

**DOI:** 10.7759/cureus.75285

**Published:** 2024-12-07

**Authors:** Taiba Trumboo, Yasmeen J Bhat, Obeid M Shafi

**Affiliations:** 1 Dermatology, Venereology and Leprosy, Government Medical College, Srinagar, IND; 2 Clinical Informatics, University of Arkansas for Medical Sciences, Little Rock, USA

**Keywords:** clinical dermatology, dermoscopy, genital ulcer, irritant contact dermatitis, jacquet’s erosive dermatitis, polymorphic vascular pattern

## Abstract

Jacquet's erosive dermatitis (JED) is a severe irritant dermatitis characterized by erosive genital and perianal lesions, often misdiagnosed due to overlapping clinical features. This case report presents two adult cases of JED with distinct clinical and dermoscopic findings. Dermoscopy revealed a characteristic polymorphic vascular pattern, including short linear, curved, coiled, dotted, and globular vessels, aiding in the diagnosis and differentiation from similar conditions. These findings highlight dermoscopy's utility as a non-invasive diagnostic tool for JED, potentially reducing the need for biopsy.

## Introduction

Jacquet's erosive dermatitis (JED) is a severe form of irritant contact dermatitis primarily affecting the anogenital region. It is characterized by papulo-erythematous and erosive lesions and is considered a more erosive variant of diaper dermatitis. While more common in children, JED can also occur in adults [1]. Clinically, JED can mimic other granulomatous and venereal diseases affecting the genitalia, and histopathological findings are nonspecific [2]. This case report presents two adult cases of JED, highlighting the dermoscopic features of this condition.

## Case presentation

Case 1

**Figure 1:**
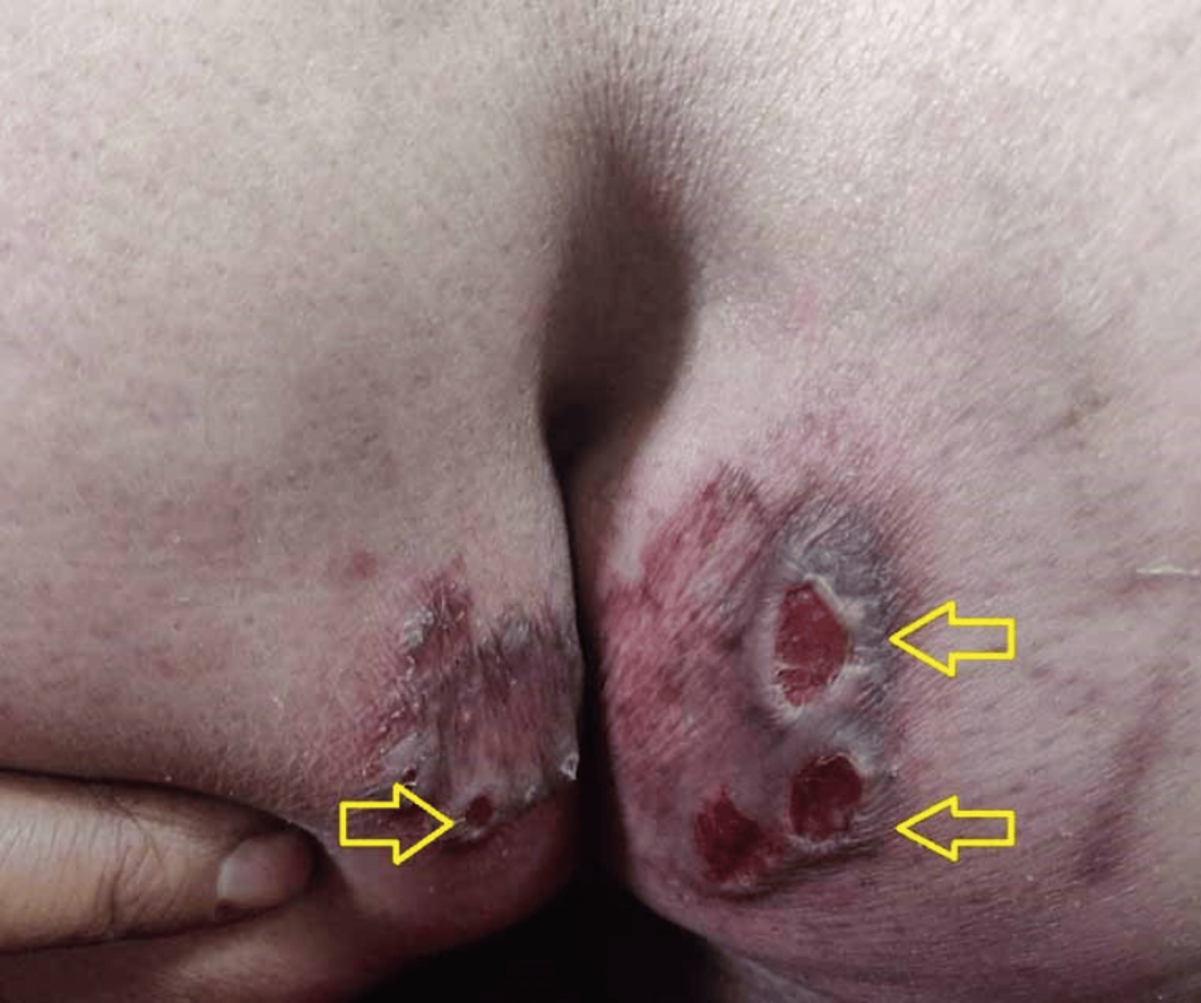
Multiple punched-out ulcers with raised edges, hyperpigmented borders, and pale granulation tissue (yellow arrows) on the gluteal area of a 65-year-old woman

A 65-year-old woman with diabetes and urinary incontinence presented with a one-year history of multiple pruritic and painful lesions on her buttocks. Examination revealed multiple well-defined, punched-out ulcers with raised edges, hyperpigmented borders, and indurated bases. The ulcer floors contained pale granulation tissue and serous discharge. The largest ulcer measured 5 x 4 cm. The gluteal and intergluteal areas were predominantly affected (Figure 1), and there was no regional lymphadenopathy.

**Figure 2:**
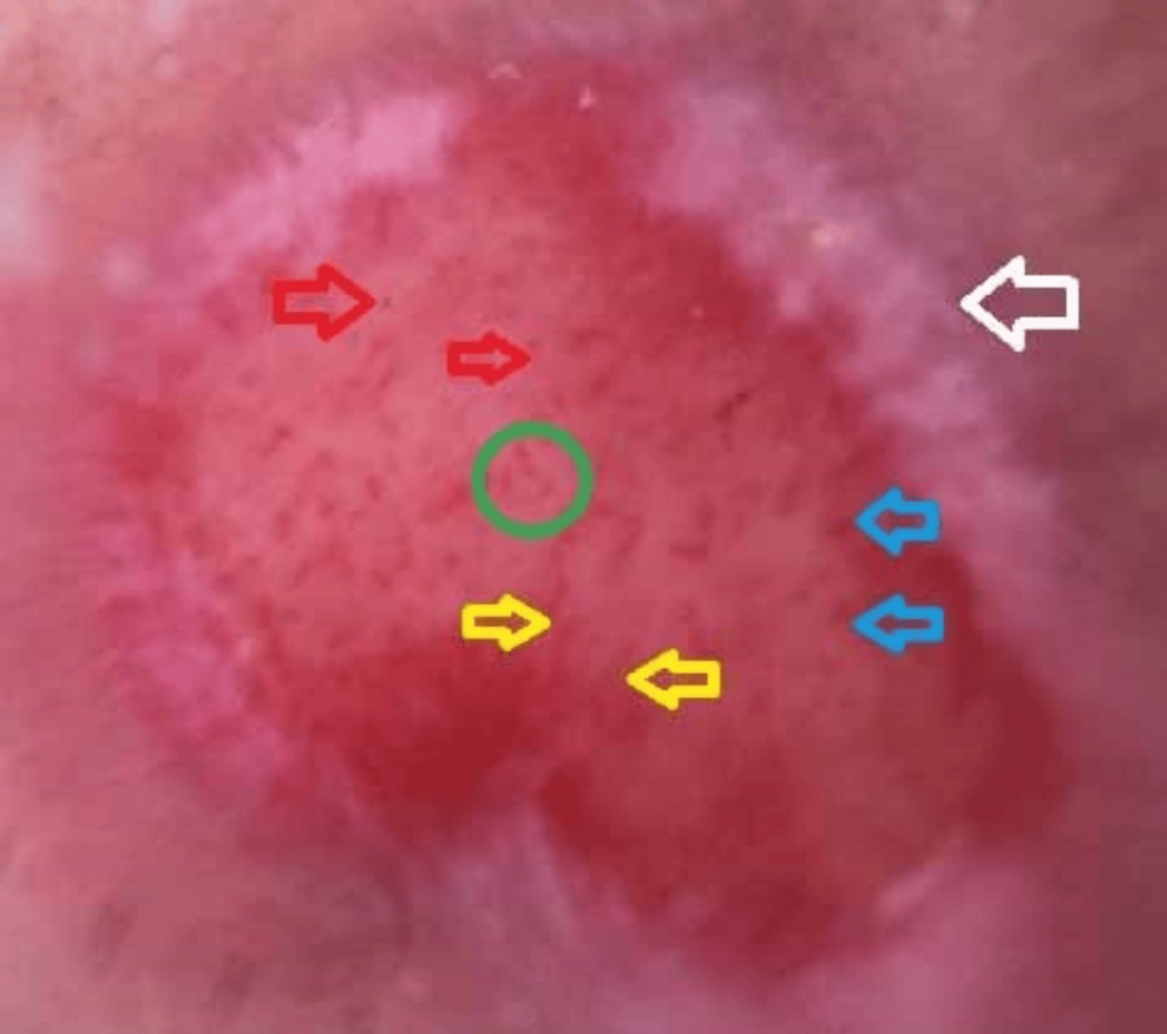
Dermoscopy (handheld, non-polarized 10x) showing beefy red areas, linear vessels (yellow arrow), dotted vessels (red arrow), globular vessels (blue arrow), curved vessels (green circle), and peripheral white areas (white arrow)

Serologic tests for syphilis were negative, and a Tzanck smear was unremarkable. Dermoscopy showed beefy red areas with curved, linear, dotted, and globular vessels, surrounded by peripheral white homogeneous areas (Figure 2). Based on these findings, a diagnosis of JED was made.

Case 2

**Figure 3:**
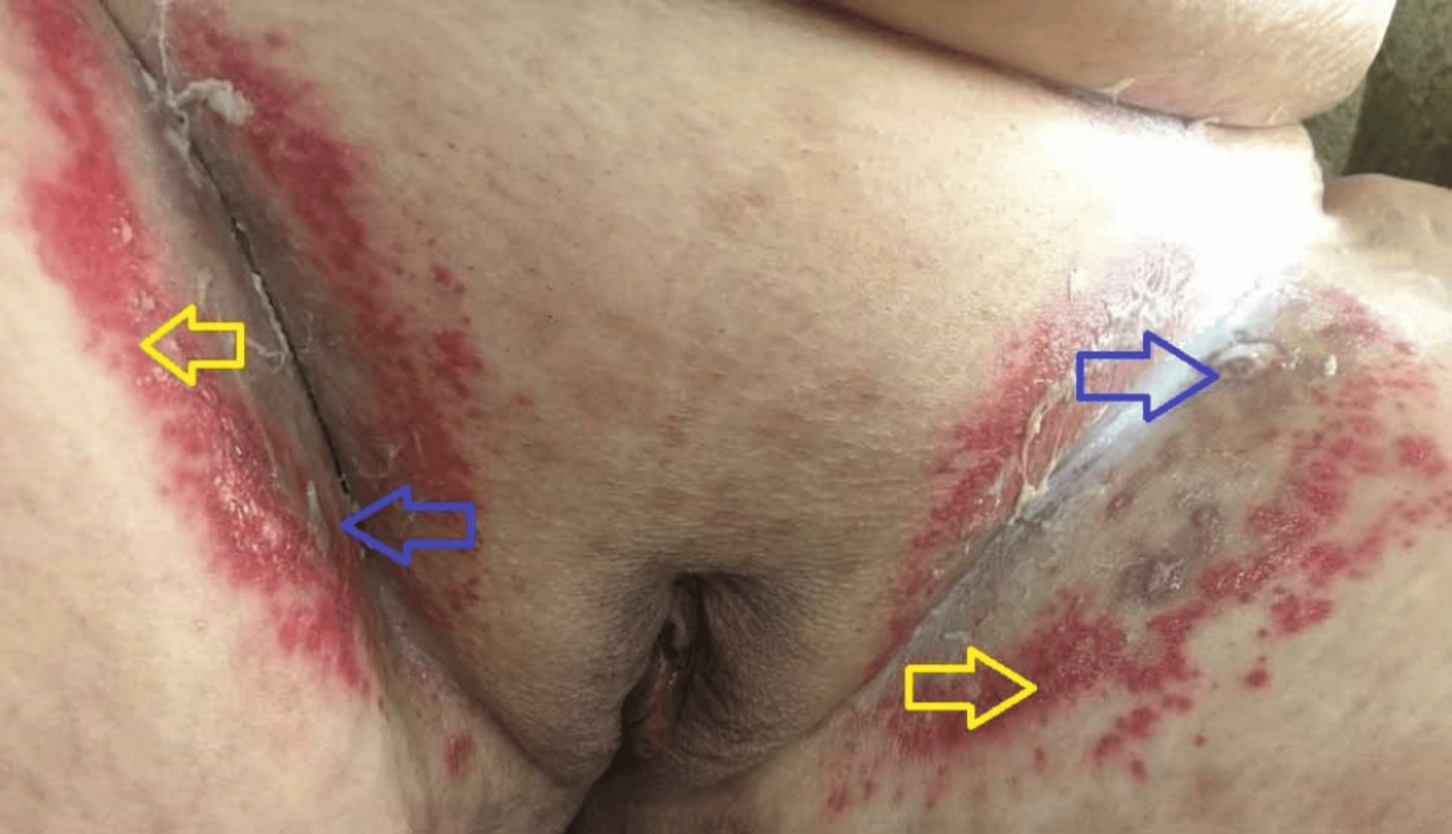
Multiple punched-out ulcers (blue arrows) surrounded by erythematous papules (yellow arrows) on the inner thighs of a 70-year-old female patient

A 70-year-old obese woman with fecal and urinary incontinence presented with four months of pruritus and shallow ulceration in the intertriginous areas. She had been using over-the-counter topical steroids and antifungals. Examination revealed multiple punched-out ulcers (2-5 mm) with raised edges and pale granulation tissue on the inner thighs, surrounded by erythematous papules (Figure 3).

**Figure 4:**
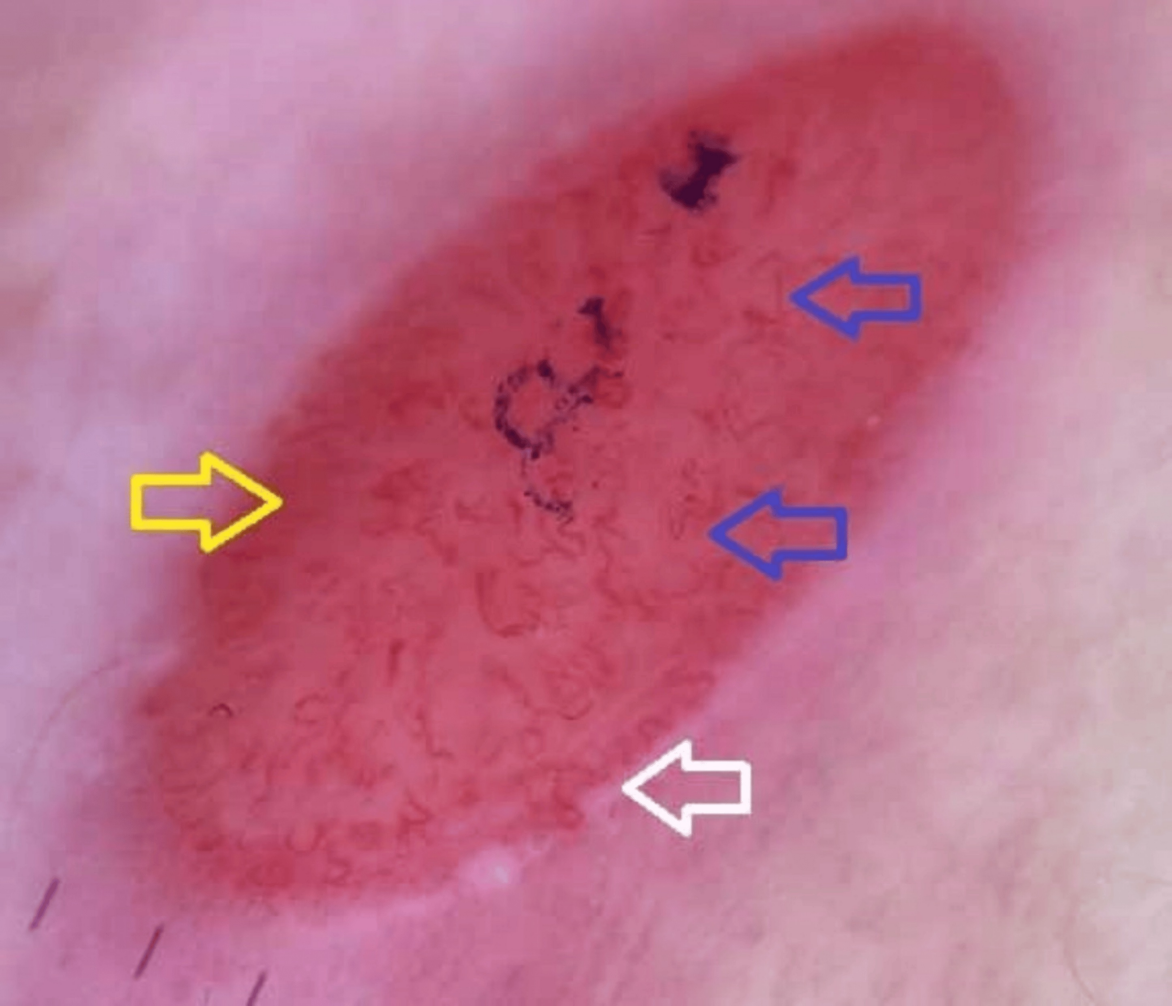
Dermoscopy (handheld, non-polarized, 10x) demonstrating short linear vessels (yellow arrow) and curved vessels (blue arrow) on a red background with a peripheral whitish rim (white arrow)

The white borders of the lesions were positive for Candida on potassium hydroxide preparation. A Tzanck smear and syphilis serology were negative. Dermoscopy showed an erythematous background with short linear and curved vessels surrounded by a whitish rim (Figure 4). A diagnosis of JED with secondary candidiasis was made.

## Discussion

JED is characterized by punched-out erosions or ulcerations with crater-like borders, often resulting from a combination of warmth, urine, moisture, feces, and secondary infection [3]. Histopathological findings are non-specific. Dermoscopy reveals a polymorphic vascular pattern: short linear, curved, coiled, dotted, and globular vessels indicative of neoangiogenesis, along with a beefy red background corresponding to hyperproliferative fibroblasts within the underlying granulation tissue [4].

The differential diagnosis for JED includes granuloma gluteale adultorum, perianal pseudo-verrucous papules and nodules, condyloma lata, condyloma acuminata, genital herpes, cutaneous Crohn’s disease, and intertriginous candidiasis (Table 1). Dermoscopy can be a valuable tool in differentiating JED [5,6].

**Table 1:** Clinical and dermoscopic differentiation of Jacquet's erosive dermatitis

Condition	Clinical features	Dermoscopic features
Jacquet's erosive dermatitis	Punched-out erosions or ulcers with crater-like borders	Polymorphic vascular pattern: short linear, curved, coiled, dotted, and globular vessels
Granuloma gluteale adultorum	Erythematous, papulonodular, or papillomatous erosive plaques	Exophytic papillae, white round areas surrounded by red-to-pink areas with a serrated white border
Perianal pseudo-verrucous papules and nodules	Shiny, moist, bright red, hard flat-topped papules and nodules that can ulcerate	White structureless areas with reticular lines
Condyloma lata	Macerated, flat, moist, wart-like papules or plaques	Well-defined white spots on a milky-red central area
Condyloma acuminata	Flat or pedunculated vegetative papules or plaques	Punctate, annular, dendritic, curved, and hairpin-like vessels
Genital herpes	Painful grouped vesicles, possible ulceration, and tender lymphadenopathy	Polylobular white structures and central brown dots corresponding to multi-nucleated giant cells
Intertriginous candidiasis	Bright red plaques with satellite papules and pustules	Cottage cheese-like structures and blurry linear vessels

Treatment for JED involves frequent diaper changes, absorbent gel-lined diapers, barrier agents like petrolatum/zinc oxide ointments, and addressing underlying factors such as incontinence. Topical antifungal or antibacterial agents may also be helpful in managing inflammation [2].

## Conclusions

JED in adults presents with distinct features that can be effectively identified using dermoscopy. The two cases highlighted in this article underscore the characteristic polymorphic vascular pattern observed in JED, which includes short linear, curved, coiled, dotted, and globular vessels. These dermoscopic findings are instrumental in differentiating JED from other granulomatous and venereal diseases affecting the genital and perianal regions.

The use of dermoscopy as a non-invasive diagnostic tool aids in accurately diagnosing JED and potentially reduces the need for invasive biopsy procedures. Recognizing the unique dermoscopic patterns associated with JED can facilitate timely and appropriate management. Future studies should explore the broader applicability of these findings in diverse patient populations.
